# Forced co-expression of IL-21 and IL-7 in whole-cell cancer vaccines promotes antitumor immunity

**DOI:** 10.1038/srep32351

**Published:** 2016-08-30

**Authors:** Yang-Zhuo Gu, Chuan-Wen Fan, Ran Lu, Bin Shao, Ya-Xiong Sang, Qiao-Rong Huang, Xue Li, Wen-Tong Meng, Xian-Ming Mo, Yu-Quan Wei

**Affiliations:** 1 State Key Laboratory of Biotherapy and Cancer Center, West China Hospital, Sichuan University, and Collaborative Innovation Center for Biotherapy, Chengdu, Sichuan 610041, PR China; 2 Department of Gastrointestinal Surgery, West China Hospital, Sichuan University, Chengdu, Sichuan 610041, PR China; 3 Institute of Digestive Surgery, West China Hospital, Sichuan University, Chengdu, Sichuan 610041, PR China; 4 Laboratory of Stem Cell Biology and Department of Pediatric Surgery, State Key Laboratory of Biotherapy, West China Hospital, Sichuan University, and Collaborative Innovation Center for Biotherapy, Chengdu, Sichuan 610041, PR China

## Abstract

Genetic modification of whole-cell cancer vaccines to augment their efficacies has a history of over two and a half decades. Various genes and gene combinations, targeting different aspects of immune responses have been tested in pursuit of potent adjuvant effects. Here we show that co-expression of two cytokine members of the common cytokine receptor γ-chain family, IL-21 and IL-7, in whole-cell cancer vaccines boosts antitumor immunity in a CD4^+^ and CD8^+^ T cell-dependent fashion. It also generates effective immune memory. The vaccine-elicited short-term effects positively correlated with enhanced infiltration of CD4^+^ and CD8^+^ effector T cells, and the long-term effects positively correlated with enhanced infiltration of effector memory T cells, especially CD8^+^ effector memory T cells. Preliminary data suggested that the vaccine exhibited good safety profile in murine models. Taken together, the combination of IL-21 and IL-7 possesses potent adjuvant efficacy in whole-cell vaccines. This finding warrants future development of IL-21 and IL-7 co-expressing whole-cell cancer vaccines and their relevant combinatorial regimens.

Vaccination with irradiated tumor cells that are genetically modified to express genes targeting different aspects of immune responses to promote antitumor immunity has been a focus in the field of tumor immunotherapeutics for decades^1^,^2^,^3^. Cancer vaccines are somewhat different from conventional vaccines, they are meant to treat cancer in most cases, rather than to prevent the onset of cancer. Therefore, immediate effectiveness is viewed as a priority. However, memory immunity should never be neglected, since long-term immunosurveillance and effective response to recurrent disease are also key to prolonged survival. Memory is an essential feature of adaptive immunity, and T cells play uniquely important part in adaptive immunity against cancer. Various signals stimulate T cell to boost the potency of adaptive immune responses, a subset of which is conducted by common cytokine receptor γ-chain family cytokines, comprising IL-2, IL-4, IL-7, IL-9, IL-15 and IL-21. Their receptors, sharing a common γ subunit, transduce signals through the Jak-STAT pathway among others, on binding to their respective ligands. Different receptors preferentially activate different subsets of STATs, which bind different cis-acting elements, thus assume diverse functions. The differential expression patterns of these receptors on T cells, as well the balance between different activated STATs, along with other factors, dictate the outcome of T cell responses. IL-21 receptor, expressed on naïve, effector and memory T cells, albeit at varied levels, signals mainly through STAT3, which is a distinctive bias from other members of this receptor family. While IL-7 receptor, expressed on naïve and memory T cells, almost absent on effector T cells though, signals mainly through STAT5^4^,^5^. IL-21 is mainly produced by activated CD4^+^ T cells. By promoting a memory phenotype in activated T cells and suppressing regulatory T cells (Tregs), it exhibits the ability to strengthen T cell response^4^,^6^,^7^,^8^,^9^,^10^. IL-7 is mainly produced by stromal cells and considered to be present in limiting amounts *in vivo*^11^. It has been shown to promote the survival and proliferation of naïve and memory T cells, thereby increasing the specificity repertoire and improving the capability of T cells to react to weak antigens^12^,^13^,^14^,^15^,^16^. Moreover, accumulating data point to a synergizing role of IL-21 with IL-7 in boosting immunity^17^,^18^,^19^.

In this study, adjuvant activity of the IL-21 and IL-7 combination was tested in transplanted murine tumor models using lentivirally transduced whole-cell vaccines. Antitumor effects were evaluated in both prophylactic and therapeutic settings. Memory responses were also assayed. We investigated which immune cell compartment(s) played important roles in the antitumor immunity generated by the vaccine, and what changes taking place in the tumor microenvironment contributed to the immunity. In addition, the vaccine formulation were examined for safety concerns in murine models.

## Results

### Generation of vaccine cell lines and verification of cytokine production

**Figure 1:**
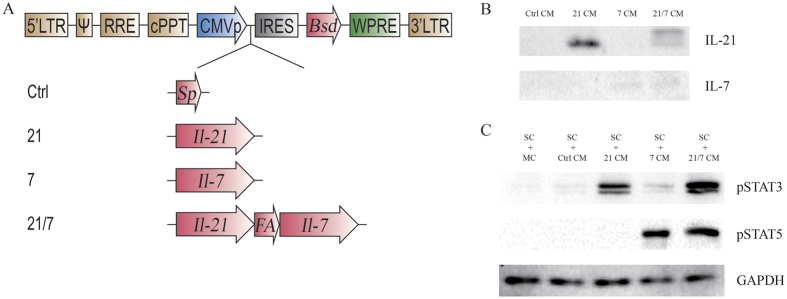
Establishment of vaccine cell lines by lentiviral transduction. (**A**) Lentiviral constructs used in this study. LTR, long terminal repeat. Ψ, packaging signal. RRE, Rev response element. cPPT, central polypurine tract. CMVp, CMV promoter. IRES, internal ribosome entry site. Bsd, blasticidin resistance gene. WPRE, woodchuck hepatitis virus posttranscriptional regulatory element. Sp, Signal peptide of IL-7. FA, furin cleavage site and P2A peptide. (**B**) Western blot analysis of secreted IL-21 and IL-7 in vaccine cell-conditioned media. Note that the two bands of IL-21 in the 21/7 CM lane represented two possible cleavage products: cleavage at furin site resulted in IL-21 + 4AA, while cleavage at P2A site resulted in IL-21 + 25AA. CM, conditioned medium. AA, amino acids. (**C**) Western blot analysis of STAT proteins activated in response to secreted IL-21 and IL-7. SC, splenocytes. MC, medium control. Full-length blots are presented in Supplementary Figure S1.

As shown in Fig. 1A, four lentiviral vectors, in which transgenes are driven by CMV immediate early promoters, were constructed. Transgenes included Il-21 (hereafter 21), Il-7 (hereafter 7), Il-21 fusion with Il-7 via a self-cleavable furin-P2A linker (hereafter 21/7)^20^,^21^, as well as Il-7 signal peptide coding sequence, serving as a control (hereafter Ctrl). Respective lentiviruses were packaged in 293T cells from these vectors, and used to transduce target tumor cell lines. Transduction efficiency were adjusted to <10%, so that the majority of transduced cells harbor only one copy of transgene^22^. The cells were selected for blasticidin resistance to establish vaccine cell lines. These B16F10 or CT26-derived cell lines were named in abbreviated formats as “16-trangene” or “26-transgene” hereafter.

Cytokine production were confirmed by western blot analysis of vaccine cell-conditioned media (Fig. 1B). The functionality of secreted cytokines were validated in murine splenocytes by prominent induction of phosphorylated STAT3 (Tyr705) and phosphorylated STAT5 (Tyr694) (Fig. 1C), characteristic of IL-21 and IL-7 signaling, respectively.

### Vaccination with IL-21 and IL-7 co-expressing cells protects mice from tumor challenge in a prophylactic B16F10 model

**Figure 2:**
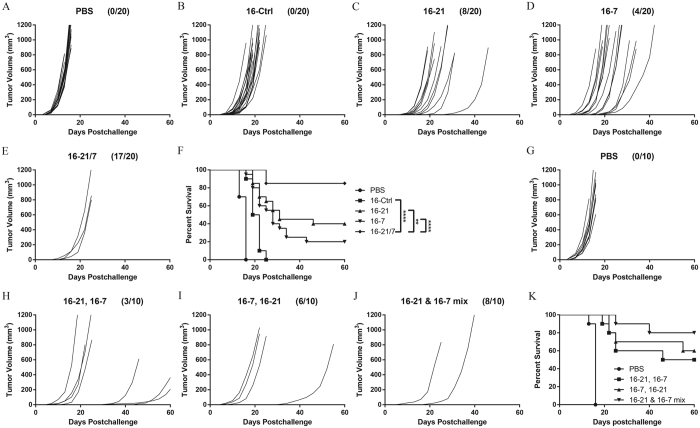
Vaccination with IL-21 and IL-7 co-expressing B16F10 cells prevented tumorigenesis of B16F10 melanoma in prophylactic setting. (**A–E**) Individual tumor growth curves of B16F10 tumors. Mice were vaccinated s.c. on day −14 and day −7 with 10^6^ irradiated tumor cells, then challenged c.l. on day 0 with 10^5^ viable B16F10 cells. Fractions in the parentheses indicate the proportion of tumor-free mice in totals. Data were pooled from two independent repeats. (**F**) Cumulative survival curves of two independent repeats. **P < 0.01. ****P < 0.0001. (**G–J**) Individual tumor growth curves of B16F10 tumors. Mice were vaccinated s.c. on day −14 and day −7 with PBS or 10^6^ irradiated tumor cells following one of the following regimens. 1) prime with 16–21 cells, boost with 16-7 cells in (**H**). 2) prime with 16-7 cells, boost with 16–21 cells in (**I**). 3) prime and boost with a mixture of 5 × 10^5^ 16–21 and 5 × 10^5^ 16-7 cells in (**J**). mice were then challenged c.l. on day 0 with 10^5^ viable B16F10 cells. Fractions in the parentheses indicate the proportion of tumor-free mice in totals. Data were pooled from two independent repeats. (**K**) Cumulative survival curves of two independent repeats.

To assess the contribution of IL-21 and IL-7 to antitumor immunity as potential vaccine adjuvants, transduced B16F10 melanoma cell lines expressing either of the two cytokines alone or together were put to test in a prophylactic B16F10 model, along with the control cell line. Mice were subcutaneously (s.c.) primed with 10^6^ lethally irradiated vaccine cells or PBS only, boosted one week later, and then challenged contralaterally (c.l.) with 10^5^ viable B16F10 cells. 85% of mice in the 16-21/7 group stayed tumor-free, while only 40% and 20% of mice in the 16–21 group and the 16-7 group were immune to lethal tumor challenge (Fig. 2C–E), which suggested a synergy between IL-21 and IL-7 when co-expressed in whole-cell vaccines. Mice of the Ctrl group and PBS group all developed tumors several days after challenge, showing very limited antitumor efficacy (Fig. 2A,B). Vaccination of 16-21/7 cells significantly improved the survival of mice, compared to other formulations (Fig. 2F).

To further determine whether the co-presence of the two cytokines in the vaccine formulation is a necessity for the generation of an optimal immune protection, mice were vaccinated with 16–21 and 16-7 cells together or in temporally and spatially separated fashions (Fig. 2H–J). In all three regimens, mice were given a total of 10^6^ 16–21 cells and 10^6^ 16-7 cells. Administration of 16–21 and 16-7 cells in mixture outperformed other regimens, yielding more tumor-free mice and improved survival (Fig. 2K).

These data demonstrated that whole-cell vaccine co-expressing IL-21 and IL-7 could elicit efficacious antitumor responses in a prophylactic setting.

### Vaccination with IL-21 and IL-7 co-expressing cells inhibits growth of established tumor in therapeutic models

**Figure 3:**
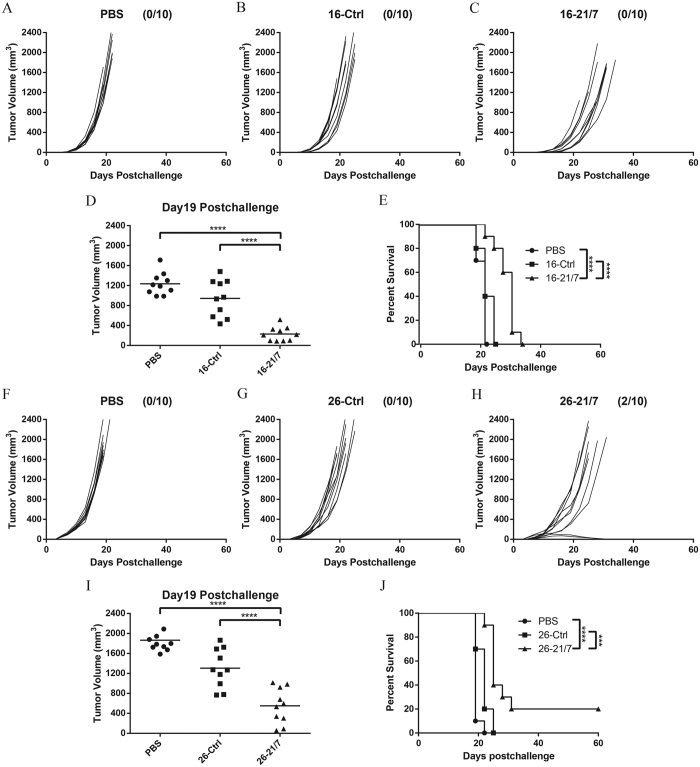
Vaccination with IL-21 and IL-7 co-expressing cells inhibited tumor progression in two therapeutic models. (**A–C**) Individual tumor growth curves of B16F10 tumors. Mice were challenged s.c. on day 0 with 5 × 10^4^ viable B16F10 cells, then vaccinated c.l. on day 3 with two doses of 10^7^ irradiated tumor cells. Data were pooled from two independent repeats. (**D**) Cumulative tumor volumes on day 19 of two independent repeats. Horizontal bars represent means. (**E**) Cumulative survival curves of two independent repeats. (**F–H**) Individual tumor growth curves of CT26 tumors. Mice were challenged s.c. on day 0 with 10^5^ viable CT26 cells, then vaccinated c.l. on day 3 with two doses of 10^7^ irradiated tumor cells. Fractions in the parentheses indicate the proportion of cured mice in totals. Data were pooled from two independent repeats. (**I**) Cumulative tumor volumes on day 19 of two independent repeats. Horizontal bars represent means. (**J**) Cumulative survival curves of two independent repeats. ***P < 0.001. ****P < 0.0001.

The efficacy of IL-21 and IL-7 co-expressing cells were further verified against established tumors. Mice were first challenged s.c. with 5 × 10^4^ viable B16F10 cells, two doses of 10^7^ irradiated transduced B16F10 cells were c.l. administrated three days later. Treatment with 16-21/7 cells retarded growth of B16F10 tumors (Fig. 3A–C), resulting in significantly smaller tumor burdens on day 19 postchallenge (Fig. 3D), as well as significantly prolonged survival of challenged mice (Fig. 3E).

In addition to weakly immunogenic B16F10 melanoma in C57BL/6 mice, the vaccines were also tested in the treatment of moderately immunogenic CT26 colon carcinoma in Balb/c mice^23^. Mice were first challenged s.c. with 10^5^ viable CT26 cells, two doses of 10^7^ irradiated transduced CT26 cells were c.l. administrated three days later. Treatment with 26-21/7 cells elicited more prominent responses in CT26 model, retarded the growth of CT26 tumors (Fig. 3F–H), and registered complete regression of established tumor in two cases, resulting in significantly smaller tumor burdens on day 19 postchallenge (Fig. 3I), as well as significantly prolonged survival of challenged mice (Fig. 3J).

These results suggested that vaccination of tumor cells expressing both IL-21 and IL-7 efficiently inhibited tumor growth in therapeutic models.

### Antitumor immunity elicited by IL-21 and IL-7 co-expressing tumor cell vaccine relies heavily on CD4^+^ and CD8^+^ T cells

**Figure 4:**
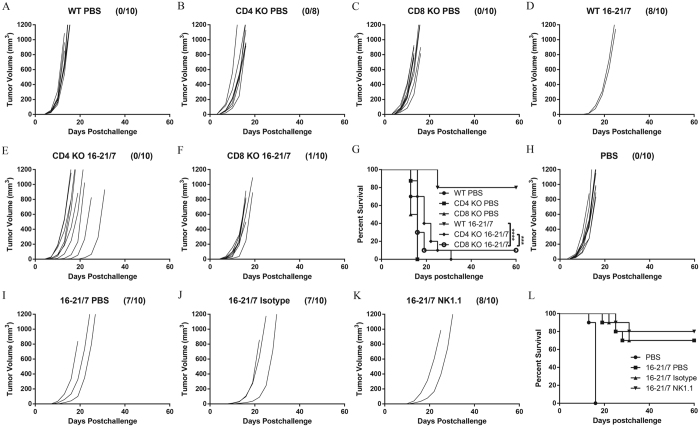
Antitumor efficacies of IL-21 and IL-7 co-expressing tumor cell vaccine depended highly on CD4^+^ and CD8^+^ T cells, rather than NK cells. (**A–F**) Individual tumor growth curves of B16F10 tumors. Wild-type, as well as CD4 KO and CD8 KO mice were vaccinated s.c. on day −14 and day −7 with 10^6^ irradiated tumor cells, then challenged c.l. on day 0 with 10^5^ viable B16F10 cells. Fractions in the parentheses indicate the proportion of tumor-free mice in totals. Data were pooled from two independent repeats. (**G**) Cumulative survival curves of two independent repeats. ***P < 0.001. ****P < 0.0001. (**H–K**) Individual tumor growth curves of B16F10 tumors. Mice were vaccinated s.c. on day −14 and day −7 with 10^6^ irradiated tumor cells, then challenged c.l. on day 0 with 10^5^ viable B16F10 cells. PBS, isotype control, or NK1.1 antibody were administrated i.p. following the protocol described in Methods. Fractions in the parentheses indicate the proportion of tumor-free mice in totals. Data were pooled from two independent repeats. (**L**) Cumulative survival curves of two independent repeats.

As T cells and/or NK cells are previously suggested as effectors in antitumor immunity induced by IL-21 vaccine and IL-7 vaccine^24^,^25^,^26^, we next set to unveil which cell compartment(s) support this immune protection conferred by vaccination with IL-21 and IL-7 co-expressing cells. Prophylactic vaccination with IL-21 and IL-7 co-expressing B16F10 cells and tumor challenge were repeated in wild-type, CD4 knockout and CD8 knockout (KO) mice, as described previously in this study. While wild-type mice showed similar (80%) protection effects to previous experiments in this study, antitumor immunity was severely compromised in both CD4 KO and CD8 KO mice, with tumor-free rate dropped sharply to 0% and 10%, respectively (Fig. 4A–F). Tumors in CD4 KO and CD8 KO mice developed faster, thus resulting in worsened survivals (Fig. 4G).

NK1.1 antibody (clone PK136), isotype control and PBS were administrated intraperitoneally (i.p.) as previously described^27^ in mice prophylactically vaccinated with the standard protocol. NK 1.1 depletion was efficient (>94%), as detected by flow cytometry. NK1.1-depleted mice exhibited no significant difference in terms of tumor-free ratio and survival from the mock-depleted counterparts (Fig. 4I–L).

These facts suggested that CD4^+^ and CD8^+^ effector T cells were important participants in the antitumor immunity triggered by IL-21 and IL-7 co-expressing tumor cell vaccine, while NK cells are dispensable in such a process.

### Vaccination with IL-21 and IL-7 co-expressing tumor cells increases tumor-infiltrating CD8^+^ and CD4^+^ effector T cells as well as effector/regulatory cell ratios

**Figure 5:**
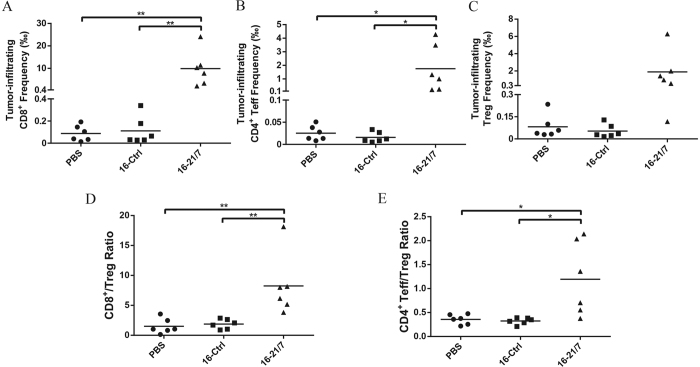
Vaccination with IL-21 and IL-7 co-expressing tumor cells enhanced CD8^+^ and CD4^+^ effector T cell infiltration in tumors and raised infiltrating effector/regulatory cell ratios. (**A–C**) Frequencies of CD8^+^, CD4^+^ effector and regulatory cells in total cells of B16F10 tumors generated by challenge s.c. with 3 × 10^6^ viable cells at day 0. Data were pooled from two independent repeats. (**D,E**) CD8^+^/Treg and CD4^+^ Teff/Treg ratios in B16F10 tumors. Data were pooled from two independent repeats. Horizontal bars represent means. *P < 0.05. **P < 0.01.

As elucidated previously, CD4^+^ and CD8^+^ T cells take center stage in bolstering the antitumor immunity elicited by IL-21 and IL-7 co-expressing tumor cell vaccine. In clinical practice, the intratumoral ratio of effector T cells versus regulatory T cells, especially the CD8^+^/Treg ratio, is a widely accepted prognostic index for many cancer types, including melanoma^28^,^29^,^30^,^31^,^32^,^33^. In light of this, we analyzed the composition of tumor-infiltrating T cells from each vaccine group with flow cytometry. Vaccination of IL-21 and IL-7 co-expressing B16F10 cells resulted in marked increased infiltration of both CD8^+^ and CD4^+^ effector (CD4^+^Foxp3^−^) T cells, especially the former population, compared to vaccination with PBS and 16-Ctrl cells (Fig. 5A,B). Treg (CD4^+^Foxp3^+^) infiltration was also increased, but not significantly different from the control groups (Fig. 5C). CD8^+^/Treg ratios were sharply raised by the IL-21 and IL-7 co-expressing vaccine, CD4^+^ Teff/Treg ratios were also significantly raised, but to a lesser extent (Fig. 5D,E).

Thus, enhanced infiltration of CD8^+^ and CD4^+^ effector T cells in tumors and altered balance between effector T cells and regulatory T cells in favor of immunity contributed to the augmented efficacy of IL-21 and IL-7 co-expressing tumor cell vaccine.

### Vaccination with IL-21 and IL-7 co-expressing cells induces memory antitumor immunity

**Figure 6:**
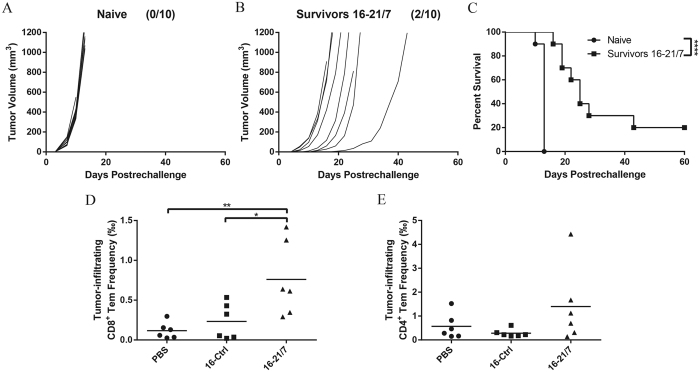
Vaccination with IL-21 and IL-7 co-expressing B16F10 cells elicited memory responses. (**A,B**) Individual tumor growth curves of B16F10 tumors. Mice remaining tumor-free were rechallenged with 2 × 10^5^ viable B16F10 cells on day 90. Fractions in the parentheses indicate the proportion of tumor-free mice in totals. Data were pooled from two independent repeats. (**C**) Cumulative survival curves of two independent repeats. ****P < 0.0001. (**D,E**) Frequencies of CD8^+^, CD4^+^ effector memory T cells in total cells of B16F10 tumors generated by challenge with 4 × 10^5^ viable cells at day 35. Data were pooled from two independent repeats. Horizontal bars represent means. *P < 0.05. **P < 0.01.

Immune memory, pivotal for prevention of both incipient and recurrent tumors, is a desired feature of vaccine-induced anti-tumor immune responses. We tested whether the vaccination was able to sustain long-lasting responsiveness. Tumor-free mice from 16-21/7 group that survived day 0 challenge with 10^5^ viable B16F10 cells were rechallenged on day 90 with higher dose of 2 × 10^5^ viable B16F10 cells, and no interventions were implemented afterwards. 20% of mice were still completely protected from tumor challenge, and the rest of mice that did develop tumors exhibited retarded tumor progression (Fig. 6A,B) and achieved significantly improved survival (Fig. 6C).

We also analyzed the presence of CD8^+^ (CD8^+^CD44^hi^ CD62L^−^) and CD4^+^ (CD4^+^FOXP3^−^CD44^hi^CD62L^−^) effector memory T cells (Tem) in tumors transplanted when the primary anti-tumor responses induced by the vaccination waned. Tumors from the 16-21/7 group were significantly more densely infiltrated with CD8^+^ Tems, compared to tumors from control groups (Fig. 6D). They were also infiltrated by higher densities of CD4^+^ Tems, but the increase was not statistically significant (Fig. 6E).

These data suggested that vaccination with IL-21 and IL-7 co-expressing cells generated immune memory. Infiltrating effector memory T cells were positively correlated with memory immunity against tumor. These facts were in accordance with the forementioned rationale that IL-21 and IL-7 support memory immune responses.

### Vaccination with IL-21 and IL-7 co-expressing tumor cells is safe in preliminary safety study

**Figure 7:**
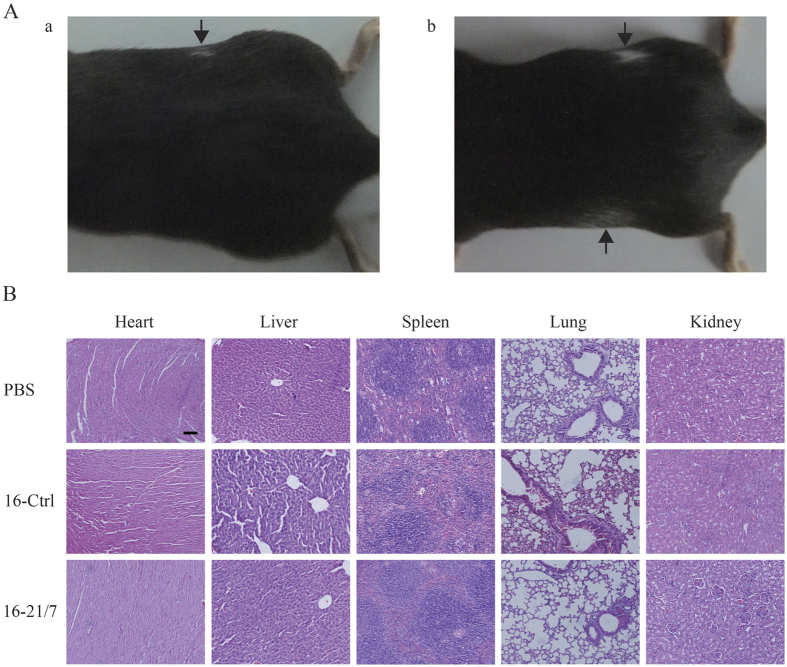
Vaccination with IL-21 and IL-7 co-expressing tumor cells exhibited good safety profile with trivial side effect. (**A**) Vaccination with IL-21 and IL-7 co-expressing tumor cells induced depigmentation. Upper arrows in (a) and (b) indicate vitiligo at challenge sites. Lower arrow in (b) indicates vitiligo at boost site. (**B**) H&E staining of vital organs of vaccinated mice. Scale bar, 100 μm.

Mice vaccinated with IL-21 and IL-7 co-expressing tumor cells exhibited no side effects on gross features, such as weight loss, ruffling of fur, behavior, etc., except for vitiligo, probably resulting from T cell reaction to shared antigens of melanoma cells and melanocytes^34^. About 40% of vaccinated mice gradually developed vitiligo of varied extent, ranging from small patch of depigmentation at challenge site (Fig. 7Aa) to large patches at both secondary vaccination site and challenge site (Fig. 7Ab). All of mice that did develop vitiligo were immune to B16F10 challenge in this study, which is consistent with currently held concept that vitiligo is a sign of good prognosis for melanoma^35^, thus this side effect of depigmentation should be more than acceptable.

Hematoxylin and eosin (H&E) staining of organ sections of vaccinated mice was also performed. No histopathological changes in internal vital organs, including the heart, liver, spleen, lung and kidney, were detected (Fig. 7B).

These preliminary data suggested that vaccination with IL-21 and IL-7 co-expressing tumor cells exhibited good safety profile, with negligible, if any, side effect.

## Discussion

In the present study, we have shown that vaccination with tumor cells co-expressing IL-21 and IL-7 elicited potent antitumor responses in both prophylactic and therapeutic tumor models. The vaccine generated immune responses that depended on both CD4^+^ and CD8^+^ T cells. Memory antitumor responses elicited by the vaccine were also validated. The vaccine-elicited short-term effects positively correlated with enhanced infiltration of CD4^+^ and CD8^+^ effector T cells, and the long-term effects positively correlated with enhanced infiltration of effector memory T cells, especially CD8^+^ Tems. Last but not the least, preliminary data suggested that the vaccine was safe in murine models.

To our knowledge, this is the first report showing that combining IL-21 with IL-7 in cancer vaccines generates enhanced antitumor immunity compared to either cytokine alone. Actually, this cytokine combination has not been described in any previous vaccine formulations. A proportion of mice receiving melanoma vaccine co-expressing IL-21 and IL-7 developed vitiligo over time, which possibly suggested that breaking of topical peripheral tolerance to self-antigens, in this case melanocyte-associated antigens, could be achieved using this new vaccine formulation. This is a phenomenon not seen with the commonly used GM-CSF-expressing vaccine alone^36^. Breaking the tolerance to self-antigens has always been a hotly pursued goal in cancer immunotherapy^37^, as cancers of nonviral etiology, covering the majority of all cancers, harbor no potential antigens other than mutated or re-arranged self-antigens, tissue-specific self-antigens and aberrantly expressed self-antigens. The present study might offer a new way to break such tolerance without causing additional problems. The sustenance of adaptive memory against cancer is also a feature worth noting for IL-21 and IL-7 co-expressing cancer vaccines, since immune memory is responsible for long-lasting protection from cancer recurrences^38^.

Previously, Croce *et al*. stated that the efficacy of IL-21-expressing whole-cell vaccine depends on CD8^+^ T cells^26^, while Ma *et al*. asserted that it is both CD8^+^ T cell and nature killer cell-dependent^24^. In addition, Schroten *et al*. found that the efficacy of IL-7-expressing whole-cell vaccine relies on NK1.1^+^ cells^25^. However, our observations indicated that IL-21 and IL-7 co-expressing whole-cell vaccine exerts its antitumor function mainly by CD4^+^ and CD8^+^ T cells, and that NK cells are dispensable in such a process. This disagreement with previous study might reflect the advantage of combining IL-21 and IL-7 in driving effective immunity by mobilizing both CD4^+^ and CD8^+^ cells.

The efficacy of whole-cell vaccines can be further boosted by introducing various cytokines, co-stimulatory molecules, pattern recognition receptor agonists, checkpoint blockers, etc. Blockade of checkpoint, namely, co-inhibitory molecules, such as CTLA-4, PD-1, LAG-3 and TIM-3, have shown great prospect in cancer treatment in both preclinical and clinical researches. By depleting and suppressing the function of regulatory T cells and augmenting the function and proliferation of effector T cells, checkpoint blocking antibodies can rescue the otherwise anergized or exhausted effector T cells, increase the infiltration of effector T cells, raise the Teff/Treg ratio in tumor microenvironment, and ultimately, bring about optimal immune responses against cancer^36^,^39^,^40^,^41^,^42^,^43^. As common cytokine receptor γ-chain family cytokines, including IL-21 and IL-7, induce the expression of PD-1 and its ligands^44^, blockade of PD-1 signaling pathway should be particularly instrumental to further improving the efficacy of IL-21 and IL-7 co-expressing vaccines presented in this study, especially in therapeutic settings. Tumor-reactive T cells, readily activated by the present vaccine formulation, might be susceptible to co-inhibition, thus are overwhelmed by the consummated immunosuppression in the microenvironment of established tumors. Checkpoint blockade should restore the function of these effector T cells, thus render the regression of established tumors more efficient. This notion should be verified in future study.

In conclusion, our data demonstrate that forced co-expression of IL-21 and IL-7 in whole-cell cancer vaccines promote antitumor immunity. The vaccine formulation stimulates CD4^+^ and CD8^+^ T cell-dependent adaptive responses, and generates immune memory. These facts warrant future development of IL-21 and IL-7 co-expressing whole-cell cancer vaccines and their relevant combinatorial regimens.

## Materials and Methods

### Animals and cell lines

Female C57BL/6 and Balb/c mice were purchased from Vital River (Beijing, China). CD4 knockout and CD8 knockout mice (C57BL/6 background) were purchased from the Jackson Laboratory (Bar Harbor, ME USA) and bred in our facility. Animal experiments were approved by the Animal Care and Use Committee of Sichuan University, and performed in compliance with the guidelines. Murine melanoma B16F10 cells and colon carcinoma CT26 cells were maintained in RPMI 1640 medium. 293T cells were maintained in DMEM medium. Both culture media were supplemented with fetal bovine serum (10%).

### Plasmid construction

Coding sequences of murine Il-21 and Il-7 genes were amplified from C57BL/6 spleen cDNA. The two fragments were either directly cloned into a lentiviral vector, pLVX-IRES-Bsd, which was derived from pLVX-IRES-ZsGreen1 (Clontech, Mountain View, CA USA) by replacing ZsGreen1 coding sequence with that of the blasticidin resistance gene, or first joined by coding sequences of a furin cleavage site and a P2A peptide, and then cloned into the same vector. IL-7 signal peptide coding sequence was cloned as forementioned to create the control vector.

### Lentiviral packaging and vaccine cell line establishment

293T cells were co-transfected with psPAX2, pMD2.G, and respective lentiviral vectors. To achieve single-copy integration of provirus in the majority of cell population, virus-containing supernatants of 293T cells were filtered through 0.45 μm filters and diluted to ensure that transduction efficiencies were <10%. The transduced cells were placed under the selection of 10 μg/mL blasticidin for 2 weeks to obtain respective vaccine cell lines.

### Western blotting

For verification of cytokine secretion, vaccine cell-conditioned media were collected and filtered through 0.45 μm filters. For validation of cytokine activity, mouse splenocytes incubated for 90 min with conditioned media were collected. Proteins from collected samples were resolved by SDS-PAGE, and transferred to PVDF membranes. The membranes were blocked and incubated with primary antibodies against IL-21, IL-7 (PeproTech, Rocky Hill, NJ USA), phospho-STAT3 (Tyr705), phospho-STAT5 (Tyr694) (Cell Signaling Technology, Danvers, MA USA) and GAPDH (Sigma-Aldrich, St. Louis, MO USA), then incubated with HRP-conjugated secondary antibodies. The blotted proteins were visualized by chemiluminescence detection.

### Animal experiments

Vaccine cells were collected, washed with PBS and lethally irradiated (100 Gy) to make whole-cell vaccines. In the prophylactic setting, C57BL/6, CD4 KO, or CD8 KO mice were vaccinated s.c. with 10^6^ irradiated B16F10 vaccine cells twice at a one week interval, and then challenged c.l. with 10^5^ viable B16F10 cells one week after the last vaccination. For NK cell depletion, the mice were vaccinated and challenged as forementioned, and administrated i.p. with doses of NK1.1 antibody, isotype controls (Bio X Cell, West Lebanon, NH USA) or PBS as previously described^27^. Tumor-free mice were rechallenged 90 days after the initial B16F10 challenge with 2 × 10^5^ viable B16F10 cells to assay for memory responses. In the therapeutic setting, mice were challenged s.c. with viable tumor cells, 5 × 10^4^ B16F10 cells for C57BL/6 strain, and 10^5^ CT26 cells for Balb/c strain, respectively. Three days later, mice were treated with two doses of 10^7^ irradiated vaccine cells c.l. and left without further intervention. Tumors were measured in two perpendicular dimensions for length (L) and width (W), and their volumes (V) were calculated with the following formula, V = 0.5 × L × W^2^. Tumor-bearing mice were sacrificed when either of the following criteria was met: 1) Tumor volumes exceeded 800 mm^3^ and 1600 mm^3^ for prophylaxis and therapeutics, respectively. 2) Tumors ulcerated or mice became moribund. To obtain tumors for TIL analysis, mice were challenged with 3 × 10^6^ viable tumor cells one week after the last vaccination, or with 4 × 10^5^ viable tumor cells six weeks after the last vaccination.

### TIL analysis

Tumor tissues were digested with Liberase TM and Dnase I (Roche, Basel, Switzerland) and filtered through 40 μm cell strainers. Cells were initially incubated with CD16/32 antibody to block Fcγ receptors, then stained with 1) the combination of APC-Cy7-conjugated CD45 antibody (BD Biosciences, Franklin Lakes, NJ USA), FITC-conjugated CD4 antibody and APC-conjugated CD8a antibody (BioLegend, San Diego, CA USA), or 2) the combination of FITC-conjugated CD44 antibody, PE-Cy7-conjugated CD62L antibody and APC-conjugated CD4 or CD8a antibody (BioLegend). For Treg discrimination, cells were further fixed and permeabilized (eBioscience, San Diego, CA USA) according to manufacturer’s instructions, stained with PE-conjugated anti-FOXP3 antibody (eBiosciences), and analyzed with FACSAria cytometer (BD Biosciences).

### Histopathological study

Hematoxylin and eosin-stained paraffin sections of heart, liver, spleen, lung and kidney tissues from vaccinated mice were scrutinized for possible histopathological changes.

### Statistical analysis

Data were analyzed with GraphPad Prism 6. Statistical significances were determined by the log-rank test and one-way ANOVA with Tukey *post hoc* tests. Comparisons with P < 0.05 were deemed as statistically significant.

## Additional Information

**How to cite this article**: Gu, Y.-Z. *et al*. Forced co-expression of IL-21 and IL-7 in whole-cell cancer vaccines promotes antitumor immunity. *Sci. Rep*. **6**, 32351; doi: 10.1038/srep32351 (2016).

## Supplementary Material

Supplementary Information
