# Supplementary material for: Forced co-expression of IL-21 and IL-7 in whole-cell cancer vaccines promotes antitumor immunity
Source: Sci Rep. 2016 Aug 30;6:32351. doi: 10.1038/srep32351 (PMC5004106; doi:10.1038/srep32351)
Supplement: Supplementary Information [file srep32351-s1.pdf]

Supplementary Information

Forced co-expression of IL-21 and IL-7 in whole-cell cancer vaccines promotes antitumor immunity

Yang-Zhuo Gu[1, *, †], Chuan-Wen Fan[2, 3, 4, †], Ran Lu[4], Bin Shao[1], Ya-Xiong Sang[1], Qiao-Rong Huang[4], Xue Li[4], Wen-Tong Meng[4], Xian-Ming Mo[4, *] & Yu-Quan Wei[1, *].

[1]State Key Laboratory of Biotherapy and Cancer Center, West China Hospital, Sichuan University, and Collaborative Innovation Center for Biotherapy, Chengdu, Sichuan 610041, PR China. [2] Department of Gastrointestinal Surgery, West China Hospital, Sichuan University, Chengdu, Sichuan 610041, PR China. [3]Institute of Digestive Surgery, West China Hospital, Sichuan University, Chengdu, Sichuan 610041, PR China. [4] Laboratory of Stem Cell Biology and Department of Pediatric Surgery, State Key Laboratory of Biotherapy, West China Hospital, Sichuan University, and Collaborative Innovation Center for Biotherapy, Chengdu, Sichuan 610041, PR China. †These authors contributed equally to this work. *Correspondence and requests for materials should be addressed to Y.-Z.G. (email: yangzhuo_gu@163.com), Y.-Q.W. (email: yuquanwei@scu.edu.cn) or X.-M. M. (email: xmingmo@scu.edu.cn).

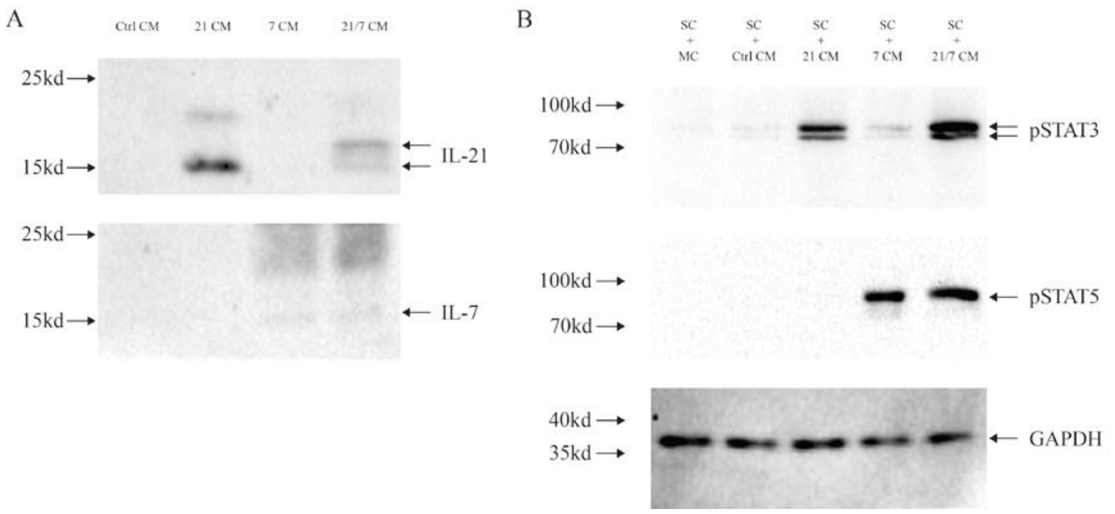

Supplementary Figure S1. Western blot analysis of IL-21 and IL-7 secreted by vaccine cell lines, and their activities in triggering STAT signaling. (A) Western blot analysis of secreted IL-21 and IL-7 in vaccine cell-conditioned media. Note that the two bands of IL-21 in the 21/7 CM lane represented two possible cleavage products: cleavage at furin site resulted in IL-21 + 4AA, while cleavage at P2A site resulted in IL-21 + 25AA. CM, conditioned medium. AA, amino acids. (B) Western blot analysis of STAT proteins activated in response to secreted IL-21 and IL-7. SC, splenocytes. MC, medium control. The cropped blots from these full-length ones are presented in Figure 1B-C.
